# Importance of History Taking and Physical Examination Before Rapid Laboratory Test for Seasonal Influenza Virus: A Single-Center Multiple Logistic Analysis

**DOI:** 10.7759/cureus.50093

**Published:** 2023-12-07

**Authors:** Yuko Tasaki, Tadafumi Yokoyama, Mari Yamamiya, Maiko Takakuwa, Tadashi Toyama, Taizo Wada, Kazuhide Ohta

**Affiliations:** 1 Pediatrics, Kanazawa University, Kanazawa, JPN; 2 Pediatrics, National Hospital Organization Kanazawa Medical Center, Kanazawa, JPN; 3 Nephrology, Kanazawa University, Kanazawa, JPN

**Keywords:** physical examination, rapid influenza diagnostic test, multiple logistic analysis, history taking, seasonal influenza

## Abstract

Background

Despite several rapid influenza diagnostic tests (RIDTs), they are predicting whether a patient has influenza before rapid testing is important. Here, we assessed factors predictive of a positive flu test via RIDTs by combining interviews and physical examination.

Methods

We analyzed the relationship between interviews and physical findings and results of RIDTs using multivariable logistic regression.

Results

Two hundred seventy-six children were enrolled throughout the 2018-2019 flu season. Accordingly, 115 patients (41.7%) were positive for flu A. Our logistic regression model identified age, body temperature, and the existence of upper respiratory symptoms as significant factors for predicting positive for RIDTs, with odds ratios (OR) of 1.17 [95% CI (confidence interval): 1.08-1.25]/+Δ1year old, 1.70 (95% CI: 1.27-2.27)/+Δ1 ℃, and 5.08 (95% CI: 2.57-10.00) for respiratory symptoms. In addition, the OR for sick contact was 7.67 (95% CI: 3.96-14.90). Our logistic regression model showed an area under the curve (AUC) of 0.84. History of vaccination was not identified as a significant factor in positive RIDTs.

Conclusions

The existence of sick contact was associated with a positive flu test via RIDTs. Although RIDTs are an easy and quick method for detecting the flu virus, we should perform the appropriate identification of cases for RIDTs by combining interviews and physical findings.

## Introduction

Seasonal influenza (flu) is an epidemic infectious disease that substantially impacts worldwide morbidity [1,2]. Even in developed countries, such as the United States, the flu is responsible for more than 200,000 hospitalizations and 3,000 to 49,000 deaths annually across all ages [3-5].

Children play a central role in the spread of the flu within communities. During annual outbreaks, the flu attack rates have remained consistently highest in children [6,7], and schools occasionally close down simultaneously [2]. Therefore, several clinicians and scientists have, for many years, continued to research methods for the prevention, early diagnosis, and treatment of the flu. Anyone from children to adults can be vaccinated annually for flu prevention. Nonetheless, studies have reported that influenza vaccination demonstrated moderate efficacy/effectiveness among young children [8].

Numerous rapid influenza diagnostic tests (RIDTs) have been developed and provided regarding the early diagnosis of the flu. Clinicians can easily perform RIDTs on febrile patients who visit their clinic during flu season.

However, all tests have certain false positive and false negative rates. RIDTs generally have a sensitivity of 50%-70% and a specificity of around 95% compared to viral culture or reverse-transcription polymerase chain reaction [2,9]. False negative results occur more often than false positive results, particularly when the prevalence of the flu is high.

Therefore, predicting patients' likelihood of being positive for the flu before performing RIDTs is important. Certain signs and symptoms can lead physicians to suspect the presence of the flu [10]. For instance, physicians would prescribe RIDTs for school children who visit the hospital for the sudden onset of high fever, a runny nose, and a cough during flu season. On the other hand, they might not perform RIDTs when an infant presenting with a fever and rash visits the hospital during non-flu season, such as in summer. However, clinicians' ability to identify potential flu cases before using RIDTs depends on their experience and skill.

The current study attempted to clarify which information acquired during medical interviews and physical examination physicians, ranging from residents to experienced clinicians, utilize to predict any presence before performing RIDTs.

## Materials and methods

Participants

We retrospectively enrolled all patients who visited the pediatric department at Kanazawa Medical Center from October 2018 to April 2019; those who underwent RIDTs were selected. The patients didn't undergo routine blood investigations and/or chest X-rays in addition to RIDTs.

We used a template for interviews and physical findings for febrile patients that any doctor can utilize for electronic medical records. The interview template was custom-created within our hospital for influenza diagnosis and treatment. Nine pediatricians (two medical directors, four medical chiefs, and three fellows) affiliated with our hospital conducted medical interviews and physical examinations and then performed RIDTs. Only patients for whom complete data on variables of interest were collected consistently across the studies were enrolled in this study.

Variables

Patient information included age (years old) and sex. We interviewed the parents of the patients and/or the patients themselves. The medical history included maximum body temperature before visiting our hospital, onset time (duration between the onset of fever and hospital visitation), history of seasonal flu virus vaccination for the current season (once or twice), an outbreak in the school and kindergarten, sick contact within 7 days of symptom onset (exposure to ill persons having symptoms of fever and acute respiratory illness, or recognized infected flu).

Patients were asked about the presence of the following symptoms and signs: headache, general malaise, chills, muscle pain, seizure, cough, rhinorrhea, sore throat, vomiting, and diarrhea. We combined cough and rhinorrhea into respiratory symptoms and vomiting and diarrhea into digestive symptoms.

The physical examination findings included body temperature at the visit, conjunctival injection (eyeball and eyelid), cervical lymphadenopathy, heart murmur, wheezes, redness of the throat, and skin erythema. Continuous variables (i.e., age and body temperature) were presented as medians and interquartile ranges (IQRs).

Rapid laboratory test for seasonal flu virus

We used either test A or test B of RIDT. Test A (Quick CHASER FluAB; MIZUHO MEDY Co. Ltd., Saga, Japan) involves immunoassays that can identify the presence of flu A and B viral nucleoprotein antigens in the nasal cavity and present the results qualitatively (positive vs. negative). Test B (Fuji dri-chem Immuno AG cartridge FluAB; FUJIFIIRM, Tokyo, Japan, and MIZUHO MEDY) involves silver amplification immunochromatography. Both Tests, A and B, are qualitative. Pediatricians selected test A or B arbitrarily.

Statistical analysis

Logistic regression analysis assessed the association between interviews, physical findings, and positive flu virus status. Odds ratios (ORs) and 95% confidence intervals (CIs) for each variable in the logistic regression model were calculated. We evaluated the predictive performance of the models by describing the receiver operating characteristic (ROC) curve and calculating the areas under the curve (AUCs), sensitivity, and specificity for flu positivity following RIDTs. All P values were two-sided, with values ≤0.05 indicating statistical significance. Statistical analyses were performed using EZR 1.45 (Saitama Medical Center, Jichi Medical University, Saitama, Japan), a graphical user interface for the R (The R Foundation for Statistical Computing, Vienna, Austria) [11].

Ethics

This study was approved by the Kanazawa Medical Center Ethics Committee (protocol number: R01-056) and Kanazawa University Hospital Ethics Committee (protocol number: 3298-1). Patients retained the right to opt-out.

## Results

Patient characteristics

**Figure 1:**
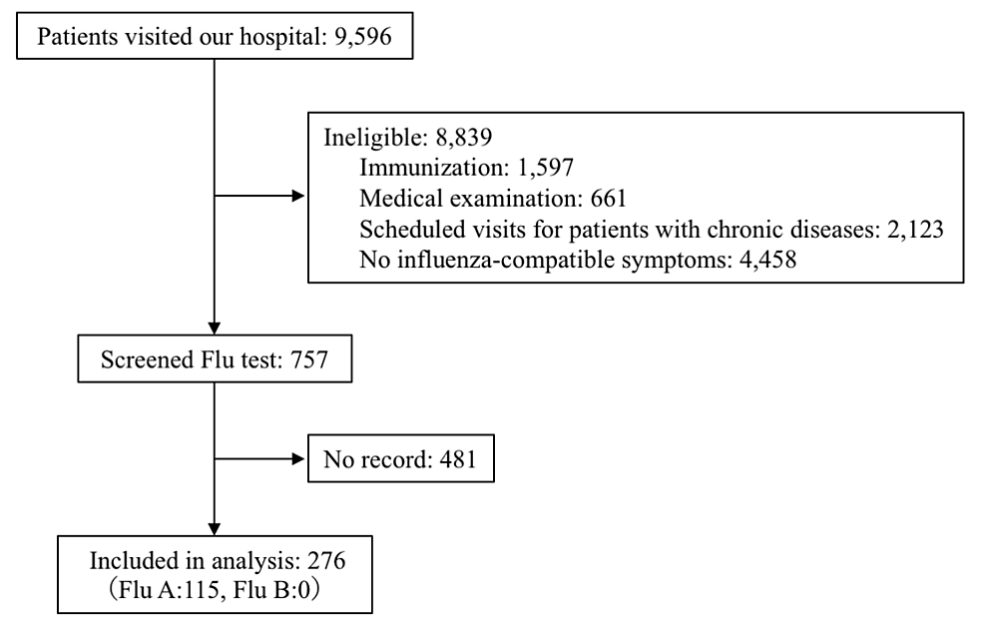
Flow diagram of patient enrollment Flu: Seasonal influenza

Nine thousand five hundred ninety-six patients visited our hospital during the study period, among whom 757 underwent flu tests. The reasons for visitation excluded health checkups, vaccination, and chronic disease. Ultimately, 276 children were included in this analysis after excluding 481 children with incomplete medical records. Among the included patients, 115 (41.7%) tested positive for flu A, whereas none tested positive for flu B (Figure 1). The general characteristics of patients enrolled herein are summarized in Table 1.

**Table 1:** Symptoms and characteristics of patients included analysis

Variable	Median [IQR], or No (%)
Age (years old)	5 [2.0-8.0]
Sex (Male)	152 (55.1)
Maximum temperature before visit	39 [38.5-39.7]
Onset: within a half day before visit	180 (65.2)
Onset: within a day before visit	238 (86.2)
Vaccination	110 (39.9)
Outbreak in the school	130 (47.1)
Sick contact	152 (55.1)
Headache	59 (21.4)
General malaise	92 (33.3)
Chills	46 (16.7)
Muscle pain	17 (6.5)
Seizure	32 (11.6)
Respiratory symptoms: Cough and/or Rhinorrhea	199 (72.1)
Cough	150 (54.3)
Rhinorrhea	162 (58.7)
Sore throat	46 (16.7)
Digestive symptoms: Vomitting and/or Diarrhea	45 (16.3)
Vomitting	37 (13.4)
Diarrhera	14 (5.1)
Temperature at visit (℃)	38.8 [38.1-39.4]
Conjunctival injection: Eye ball	28 (10.1)
Conjunctival injection: Eyelid	32 (11.6)
Cervical lymphadenopathy	10 (3.6)
Heart murmur	4 (1.4)
Wheezes	7 (2.5)
Redness of the throat	171 (62.0)
Skin erythema	10 (3.6)

The median patient age was 5.0 (IQR: 2.0, 8.0) years, and 152 (55.1%) were male. Moreover, 65.2% of the enrolled patients visited our department half a day after the onset of fever, whereas 86.2% visited after a day. Vaccination was done in 39.9% of the patients. Furthermore, 72.1% of the patients exhibited some respiratory symptoms, such as rhinorrhea and/or cough, whereas 62% showed redness of the throat.

Results of logistic regression model for Flu

We created three logistic regression models for predicting flu positivity following RIDTs (Table 2).

**Table 2:** Results of logistic regression model for influenza test positive OR: Odds ratio, CI: Confidence interval, AUC: Area under the curve

	Model 1		Model 2		Model 3	
Variables	OR (95% CI)	p-value	OR (95% CI)	p-value	OR (95% CI)	p-value
Age (+Δ1year )	1.13 (1.06-1.19)	<0.001	1.17 (1.08-1.25)	<0.001	1.13 (1.05-1.23)	0.002
Male (vs. female)	0.63 (0.38-1.03)	0.065	0.65 (0.38-1.12)	0.12	0.56 (0.31-1.03)	0.061
Body temperature (+Δ1℃)			1.70 (1.27-2.27)	<0.001	2.07 (1.49-2.86)	<0.001
Respiratory symptoms			5.08 (2.57-10.00)	<0.001	4.91 (2.36-10.20)	<0.001
General malaise			2.02 (1.11-3.69)	0.022	2.18 (1.12-4.25)	0.022
Sick contact					7.67 (3.96-14.90)	<0.001
Vaccination					0.85 (0.46-1.59)	0.61
AUC (95%CI)	0.67 (0.61 - 0.73)		0.77 (0.71- 0.82)		0.84 (0.80 - 0.89)	

Model 1, the basic model, included age and sex, whereas model 2 included physical symptoms (body temperature, respiratory symptoms, and general malaise) in addition to those in model 1. In model 3, medical history (sick contact and presence or absence of vaccination) was added to the variables in model 2.

**Figure 2:**
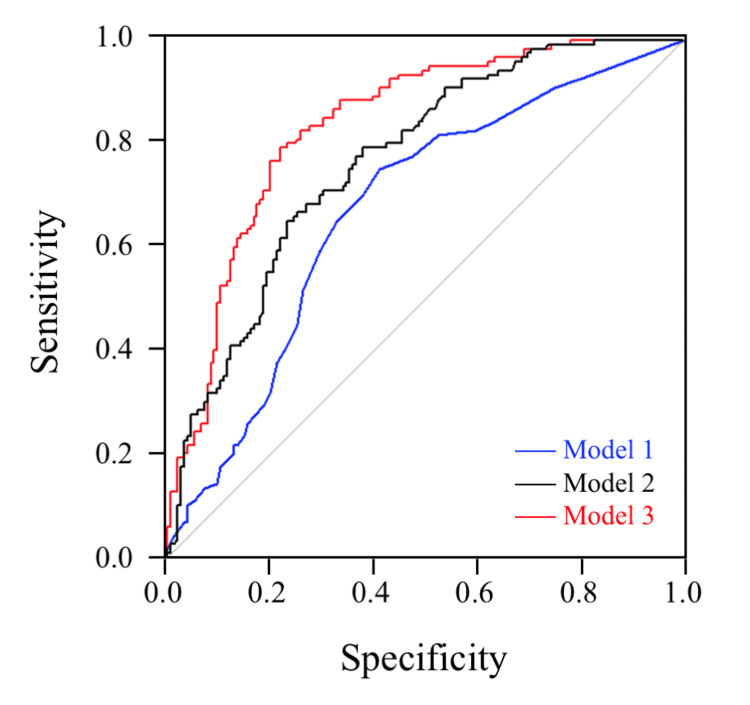
Comparison of ROC curve for Model 1～3 Model 1: Age and male. Model 2: Body temperature, respiratory symptoms, and general malaise add on Model 1. Model 3: Sick contact and vaccination add on Model 2.

Our findings showed that including interviews and physical symptoms increased the AUC. Model 3 showed the highest AUC among the models (Figure 2).

The OR for sick contact was 7.67 (95% CI: 3.96-14.90), the highest among the studied variables. However, our findings showed that vaccination was not a significant factor for predicting flu positivity after RIDTs.

## Discussion

The current study found that combining interviews and physical examination was important for predicting influenza test results [12]. While similar studies have been available to date, they have focused solely on symptoms, with only a few studies having analyzed a combination of interviews and physical examination [12,13].

During our examination, various variables had been investigated. In particular, we built a model focusing on previously reported variables [6]. Accordingly, as previously reported, we found that older age and higher body temperature were associated with a higher OD for positive RIDT results for the flu. However, our findings showed that general malaise was not associated with positive RIDT results for the flu. Nonetheless, we found that sick contact increased the likelihood of testing positive for the flu following RIDTs and that an epidemic outbreak in schools was not a significant factor (data not shown).

Interestingly, vaccination history based on interviews also did not appear to affect the results of RIDTs. Vaccinated patients might not undergo RIDTs, given the physicians' preconception that vaccinated patients were not infected or had mild symptoms. Hence, it is impossible to conclude that vaccinated patients did not test positive for the flu. We suggest that flu tests be performed when any indication of the flu is identified after an interview and physical examination, regardless of flu vaccination status.

Although RIDTs are an easy and rapid method for detecting the flu, they have some negative effects. First, collecting nasal swabs can be painful for children. Second, facilities that perform drive-through tests sometimes only emerge after the flu becomes more prevalent [14]. Considering that Japan has a universal health insurance system, every citizen has easy access to medical examinations [10,12,15,16]. As such, clinics become saturated during the flu season [12]. There is a need to determine methods for predicting those who would test positive for the flu before RIDTs [10,12]. Of the patients enrolled in this study, 65.2% had undergone RIDTs within half a day of fever and 86.2% within one day. The strategy of immediately conducting RIDTs may be inefficient in terms of time and cost. Therefore, we believe that an attempt to estimate the pre-test probability of RIDTs, as demonstrated in our research findings, is important.

However, predicting flu diagnosis before RIDTs remains challenging given that influenza does not have characteristic symptoms, with most cases exhibiting so-called cold-like symptoms, such as fever, cough, and runny nose, albeit slightly stronger. Several previous studies have shown that signs and symptoms carry predictive value for predicting positive results for the flu among children [1,6]. Various symptoms can be used to discriminate the flu from other infections. For instance, symptoms of cough, high fever, nasal congestion, and myalgia have been frequently used as predictive factors [1,6,17]. Though respiratory symptoms, myalgia, malaise, and headache were good predictors, these signs were not typical of the flu [18]. Other reports have concluded that individual signs and symptoms had limited value for diagnosing the flu [19]. Although attempts to diagnose the flu from such clinical features have remained unsuccessful, enhanced clinical recognition of the flu would be of great importance [20,21], especially in settings where laboratory diagnostics are not readily available or when the number of patients exceeds the capacity for viral testing.

In particular, interviews and physical examinations may preclude the need for invasive RIDTs among infants and reduce medical expenses [12]. Our study revealed that careful interviews and examination can predict RIDT results, highlighting their importance in clinical practice.

The current study has some limitations worth noting. First, it remains unclear whether the same results can be obtained for future seasonal flu, given that only one flu season has been investigated at a single center. Second, given that two types of RIDTs had been used, decisions regarding which kit to use were left to the physician's discretion, possibly introducing bias due to the difference in sensitivity between detection kits. Third, we only investigated factors that predicted positive results in patients who had undergone RIDTs and were not able to investigate those that influenced the physician's decision to perform RIDTs. Therefore, the results may not universally apply to all flu RIDTs in every situation.

Despite these limitations, our study confidently highlights the importance of conducting interviews and physical examinations before performing RIDTs. In addition, our findings suggest combining interviews and physical examinations, which are basic aspects of medical care, can predict positive results for the flu.

## Conclusions

Our study demonstrated that conducting interviews and physical examinations before administering RIDTs may predict the test results in some situations. Specifically, older age, higher body temperature, the presence of respiratory symptoms, general malaise, and sick contact were associated with positive RIDT results for the flu. Therefore, although RIDTs are an easy and quick approach to detecting the flu, we recommend performing them after estimating the pre-test probability through interviews and physical examinations.
